# Neurosarcoidosis

**DOI:** 10.2174/157015911796557975

**Published:** 2011-09

**Authors:** David Lacomis

**Affiliations:** Departments of Neurology and Pathology (Neuropathology), University of Pittsburgh School of Medicine, Pittsburgh, Pennsylvania 15213, USA

**Keywords:** Neurosarcoidosis, sarcoidosis, cranial neuropathy, meningitis, neuroendocrine dysfunction, seizures, peripheral neuropathy, myopathy.

## Abstract

Neurosarcoidosis is an uncommon but potentially serious manifestation of sarcoidosis. While the cranial nerves are most frequently affected, neurosarcoidosis can involve other nervous system tissues including the meninges, brain parenchyma (especially the hypothalamic region), spinal cord, peripheral nerve, and muscle. Diagnosis may be particularly challenging when neurosarcoidosis occurs in isolation. Diagnostic criteria usually include histologic identification of a noncaseating granuloma, supportive laboratory or imaging tests or both, and a compatible clinical course. Treatment has not been subjected to rigorous study, but corticosteroids are typically the first line of therapy and approximately half of patients have substantial benefit. For patients who are refractory to or intolerant of corticosteroid therapy, second-line agents include azathioprine, methotrexate, cyclosporine, cyclophosphamide, mycophenolate, and even cranial irradiation. The combination of infliximab and mycophenolate mofetil is under study as well. Treatment options will likely evolve as well-designed studies are undertaken.

## INTRODUCTION

Sarcoidosis is a systemic granulomatous disease that is still of undetermined etiology [1, 2]. In the United States, the incidence ranges from 11 per 100,000 in Caucasians to 36 per 100,000 in African-Americans, and it tends to manifest prior to age 40. However, it occurs in people of all ages and races [1, 2]. The respiratory and lymphatic systems are most commonly affected [1]. When the nervous system is involved as it is in about 5-13% of cases, that involvement is termed “neurosarcoidosis” [3-6]. Neurosarcoidosis can occur either in isolation or along with other features of systemic sarcoidosis.

Neurosarcoidosis has protean manifestations and can masquerade as many other diseases. It can affect a combination of intracranial structures, such as leptomeninges, cranial nerves and hypothalamus, and it can affect the spinal cord and its coverings as well as peripheral nerve and muscle.

Spontaneous improvement or remissions occur in about 60% of patients with neurosarcoidosis [7]. The mortality rate in all forms of sarcoidosis is from 1-5% and is due to severe pulmonary, cardiac, or neurologic disease [8].

Most of our current knowledge of neurosarcoidosis comes from retrospective and autopsy series, and from studies involving patients with systemic sarcoidosis. The immunopathogenesis and treatments of neurosarcoidosis are similar to those of systemic disease, and they have not been subjected to placebo-controlled, double-blinded studies.

## EPIDEMIOLOGY OF NEUROSARCOIDOSIS

The typical mean age of onset is from 33 to 41 years, slightly later compared to other forms of sarcoidosis [4, 9-11]. About half of these patients have known generalized disease, and 30-70% present with neurologic symptoms [4, 6, 9]. Neurologic manifestations usually occur within the first two years of illness [4, 10]. In general, neurosarcoidosis, like systemic sarcoidosis, is more common among blacks. Women make up the majority in most, but not all, series. For example, in the large series reported by Stern*, et al.,* 85% of patients with neurosarcoidosis were black, and 64% were female [4]. In contrast however, a French study of 35 patients reported that 91% were Caucasian [6], and a United Kingdom study reported that 29 of 30 patients were Caucasian, and 53% were male [9].

Risk factors specific to neurosarcoidosis have not been identified. Research involving the immunopathogenic aspects of neurosarcoidosis is lacking, and it is currently felt that the inflammatory response in the nervous system is similar to that seen in other organs, including the lung. The immunopathogenesis of sarcoidosis will be discussed later.

## CRANIAL NEUROSARCOIDOSIS

Symptoms of cranial neurosarcoidosis are varied and commonly include headache, ataxia, visual disturbances, fatigue, nausea and vomiting. Others include weakness, sensory disturbances, seizures, cognitive dysfunction, eye pain, depression, aphasia, and tremor [9]. The combination of symptoms present in an individual depends on the localization of the inflammatory process.

### Cranial Neuropathies

Cranial neuropathies are the most common manifestation of neurosarcoidosis (see Table **1**) [3-7, 9, 10, 12]. Any cranial nerve (CN) may be affected, and multiple cranial nerve involvement is common. In older series, the most frequently affected CN is VII, and sometimes bilateral facial neuropathies are present [10]. In the more recent series, optic neuropathy (discussed below) is more common [9]. Involvement of CN VIII (uni- or bilaterally) can cause auditory or vestibular dysfunction [4], but it may be asymptomatic and detected by brainstem auditory-evoked responses [10]. Nerves involved in eye movement (CN III, IV, and VI) may also be affected. Olfactory involvement is somewhat uncommon and causes anosmia and impaired taste [10, 13].

Cranial neuropathies may occur because of basilar meningitis, but infiltration or compression of nerves along their course can cause their dysfunction. For example, olfactory nerve dysfunction can occur from olfactory bulb involvement as well as from involvement of the nasal mucosa [13]. In the latter instance, it can be diagnosed by nasal biopsy. In addition, some facial neuropathies probably occur from basilar meningitis, while other cases may be due to granulomatous involvement of the extracranial portion of the nerve.

### Optic Neuropathy

Optic neuropathy is uncommon in some series but more common in others (Table **1**), and it may be serious. Bilateral involvement sometimes occurs [11]. Symptoms and signs may include decreased or blurred vision, papilledema, optic nerve atrophy, retrobulbar pain, visual field defects, and pupillary abnormalities [5, 11]. There may be local granulomatous involvement of the optic nerve, but papilledema can also be caused by increased intracranial pressure from hydrocephalus or meningeal involvement. Overall, optic neuropathy is less frequent than other common ocular features of sarcoidosis including anterior uveitis, conjunctival granulomas, scleritis, episcleritis, keratitis, posterior segment disorders, and lacrimal involvement with sicca symptoms [14].

### Acute or Chronic Meningitis

Meningeal infiltration preferentially involves the basal leptomeninges. It has been reported in up to 40% of patients (Table **1**). It may manifest with cranial nerve palsies. It can lead to hydrocephalous from cerebrospinal fluid outflow obstruction, ventricular system granulomas, or choroid plexus infiltration [5]. Cerebrospinal fluid (CSF) studies --discussed in detail later-- reveal mononuclear inflammatory cells with an elevated protein. Occasionally, the glucose is low. Magnetic resonance imaging (MRI) with contrast usually reveals leptomeningeal enhancement. The course can be monophasic, chronic, or relapsing, and is usually associated with a good outcome [8].

### Hypothalamic Dysfunction, Intracranial Masses, and Encephalopathy

Hypothalamic and pituitary dysfunction are relatively common (Table **1**), usually due to subependymal granulomatous infiltration in the region of the third ventricle. Diabetes insipidus and hyperprolactinemia are the two most common endocrine manifestations along with hypogonadism [15].

Encephalopathy can occur from neuroendocrine disturbances, leptomeningeal infiltration, mass lesions, seizures, and small vessel vasculitis. Rarely, central nervous system (CNS) sarcoidosis causes arterial and venous infarcts or transient ischemic attacks [6, 16, 17].

Granulomas in or adjacent to the brain parenchyma may mimic gliomas or meningiomas [18]. Some can also cause cerebellopontine angle masses mimicking schwannomas. Masses may be asymptomatic, but they can obstruct the ventricular system and cause hydrocephalus. Others may cause seizures [15].

### Seizures

Seizures occur in 7-22% of patients with neurosarcoidosis (Table **1**). They are the presenting manifestation in about 10%, and may be generalized or focal [19]. Their etiologies include leptomeningeal infiltration with cortical irritation, parenchymal masses, metabolic disturbances related to hypothalamic dysfunction, and possibly small vessel vasculitis associated with granulomatous angiitis. Cerebrospinal fluid and electroencephalography results may correlate poorly with clinical findings [5]. The overall prognosis can be poor because of the severity of CNS disease [5]. The seizures are usually well-controlled with anticonvulsants [19].

## SPINAL CORD AND ITS COVERINGS

Neurosarcoidosis can cause arachnoiditis, cauda equina dysfunction, extra- and intra-dural, extra-medullary and intra-medullary lesions. The incidence in modern studies utilizing MRI is 15-28% [9, 11, 20]. Junger, *et al.,* reported 16 patients with intra-medullary lesions studied retrospectively. MRI and clinical findings were noted. Five patients had sarcoidosis isolated to the spinal cord, while ten had systemic disease. The median age of onset was 35. Patients had myeloradicular symptoms. MRI showed spinal cord enlargement in seven patients, atrophy in four, and focal regions of increased T2 signal in two. One patient had diffusely increased T2 signal throughout the spinal cord. There was multifocal or focal enhancement in 50% and diffuse enhancement in 1 of 12 [21].

## PERIPHERAL NEUROPATHY

Peripheral neuropathy occurs in 4-20% of patients with neurosarcoidosis (Table **1**). Subtypes include chronic sensorimotor axonal polyneuropathy, multiple mononeuropathies, sensory polyneuropathy including small-fiber neuropathy, acute inflammatory demyelinating polyneuropathy (AIDP) and chronic inflammatory demyelinating polyneuropathies [22-26]. The overall incidence of neuropathy in Oksanen’s series was relatively high at 40% due to a relatively large number of mononeuropathies. The ulnar and peroneal nerves were most commonly affected [10]. Neuropathy can occur at various stages of sarcoidosis, and it can be the initial feature. Cranial neuropathies are more commonly encountered with the AIDP and multifocal mononeuropathy forms.

**Fig. (1):**
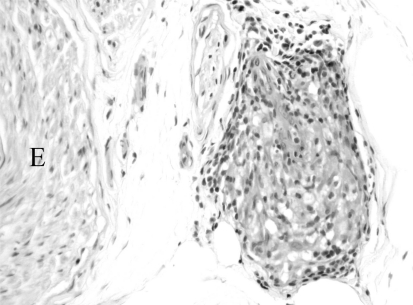
A hematoxylin- and eosin-stained paraffin section of a superficial peroneal sensory nerve biopsy specimen reveals a granuloma consisting of epithelioid histiocytes surrounded by a rim of lymphocytes. Giant cells are not seen and may not be readily apparent in most nerve granulomas. The granuloma is in the epineurium adjacent to the endoneurium (E).

The presentation depends on the type of neuropathy. Diagnosis is typically confirmed by nerve biopsy, which shows the characteristic noncaseating granulomas, (Fig. **1**). In some biopsy specimens, there is also evidence of necrotizing vasculitis or microvasculitis [24]. The mechanism of the neuropathy is often uncertain but could be due to compression by granulomas and immune factors with either axonal loss or demyelination. Vasculitis can also cause ischemic axon loss. Electrodiagnostic studies in patients with an AIDP presentation are similar to those seen in Guillain-Barré syndrome. CSF pleocytosis is more likely to be present in the patients with sarcoidosis rather than in typical AIDP, though a pleocytosis with an elevated CSF protein can also be present in HIV-associated AIDP for example [27]. A low CSF glucose would favor sarcoidosis.

Evaluation of intraepidermal nerve fiber density may be useful in confirming a diagnosis of small-fiber neuropathy when suspected and when electrodiagnostic studies do not show large-fiber neuropathic dysfunction. Subclinical involvement may also be identified by either test [28].

## MYOPATHY

Muscle involvement is commonly noted in autopsy and clinical series, and it is usually asymptomatic [29, 30]. It may be detected subclinically in up to 50% of sarcoidosis patients who undergo muscle biopsy as part of the evaluation [29]. Symptomatic muscle involvement is present in less than 1% of patients with systemic sarcoidosis. It is somewhat more common in conjunction with other features of neurosarcoidosis (Table **1**), in post-menopausal women, [31] and in the presence of erythema nodosum [29]. Its onset tends to occur later with other organ involvement [31]. Presentations include acute myopathy with a polymyositis-like presentation and chronic myopathy with muscle wasting [30]. Patients may have muscle tenderness, and sometimes nodules may be palpated [32, 33]. Diaphragm involvement is rare [34].

**Fig. (2):**
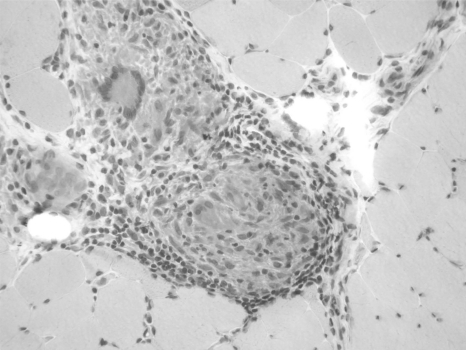
A photomicrograph of a hematoxylin- and eosin-stained paraffin section from a skeletal muscle biopsy specimen reveals a granuloma containing giant cells. It is located in the perimysium. Some lymphocytes course outside the granuloma, but the adjacent muscle fibers are mostly unaffected.

The serum creatine kinase is sometimes elevated. EMG reveals “myopathic” motor unit potentials with or without fibrillation potentials [35]. Diagnosis is confirmed by identification by noncaseating granulomas in muscle tissue. They are usually located in the perimysium, and sometimes occur in the endomysium but do not typically infiltrate myofibers distant to the granuloma (Fig. **2**).

## DIAGNOSIS

The criteria for diagnosis of neurosarcoidosis usually include a compatible clinical scenario, histologic identification of a noncaseating granuloma in any tissue, and imaging or laboratory tests supportive of the diagnosis. Zajicek *et al.* proposed diagnostic criteria with levels of certainty, and these criteria are now commonly used [20]. All categories include a clinical presentation suggestive of neurosarcoidosis and exclusion of other diagnoses. These are the criteria for *possible neurosarcoidosis*. For a *definite *diagnosis, there should also be “positive” nervous system histology. For a diagnosis of *probable neurosarcoidosis*, laboratory support (CSF or MRI) is required as well as evidence of systemic sarcoidosis (histological, Kveim test, and/or two or more indirect indicators: suggestive Gallium scan, chest imaging, or serum ACE) [20].

The approach to diagnosis is dependent on the presumed localization of the neurologic lesion and whether or not the patient has known evidence of systemic sarcoidosis [2]. Since intrathoracic disease is most common, screening for systemic sarcoidosis usually starts with a chest x-ray. Chest x-rays are abnormal in up to 90% of sarcoidosis patients, and hilar adenopathy is the most common finding. High resolution chest CT is more sensitive, especially for detecting nodules along the bronchovascular bundle and subpleural regions [36]. In addition, skin lesions should be sought. The presence of Löfgren syndrome (erythema nodosum, bilateral hilar adenopathy, fever and arthritis) is practically diagnostic of sarcoidosis. Ophthalmologic evaluation, including slit lamp exam for uveitis and other ocular findings, should also be performed. An endocrine evaluation should be undertaken if hypothalamic or pituitary dysfunction is suspected.

A minority of patients may have an elevated erythrocyte sedimentation rate. Elevations in serum alkaline phosphatase and calcium are uncommon. The serum angiotensin converting enzyme (ACE) level may be elevated in up to 65% of patients, usually in the setting of active disease, but it is insensitive [6]. The Kveim test, which utilizes intradermal injection of a single antigen from a spleen removed in 1981, is very sensitive [20], but it is not readily available.

Bronchoalveolar lavage may reveal a lymphocytosis with a high CD4:CD8 ratio, but this finding is also insensitive and non-specific [36]. Gallium-67 scanning is cumbersome and findings are nonspecific, but it may be useful in detecting a site for biopsy [6, 36]. Whole-body flourodeoxyglucose positron emission tomography may be more sensitive and useful in identifying occult granulomas, but a positive finding by itself is not diagnostic and such scanning is expensive and not widely available. Its utility has not been compared against Gallium-67 imaging [12].

Ultimately, a tissue diagnosis is required in the vast majority of patients. Common methods of biopsy would include transbronchial or endobronchial biopsies, lymph node biopsy, as well as biopsy of skin lesions [37]. Peripheral nerve, muscle, and sometimes CNS tissues are biopsied in patients with isolated neurosarcoidosis. Intraepidermal nerve fiber density analysis with punch skin biopsy may reveal reduced density scores [28], consistent with a small fiber neuropathy. Non-caseating granulomas are not expected on normal appearing skin of biopsy sites for nerve fiber density analysis.

In patients already diagnosed with sarcoidosis, additional recommended evaluations include pulmonary function testing, complete blood count, serum chemistries with calcium, liver enzymes and renal function tests, urinalysis, an electrocardiogram, and tuberculin skin test [1, 36].

### Neurosarcoidosis

Computed tomographic (CT) scans may show hydrocephalus, intracranial calcification, and enhancing nodules, [4] but they are less sensitive than MRI. MRI is very sensitive in detecting abnormalities in neurosarcoidosis, [20, 38-40] but it is nonspecific. It can detect evidence of meningeal inflammation, diencephalic involvement and parenchymal lesions in about 40%. Non-specific white matter changes are most common. In particular, periventricular T2-hyperintense lesions are often seen [11, 41, 42] and may mimic multiple sclerosis, also affecting the corpus callosum [43]. Optic nerve enlargement and enhancement also occurs in some [20]. In contrast to multiple sclerosis, linear enhancement along Virchow-Robin spaces may be more typical of neurosarcoidosis and stem from granulomatous vasculitis; nonspecific leptomeningeal and parenchymal enhancement occurred in 19% in one series [11]. In addition, parenchymal or meningeal enhancement may last longer (more than a few weeks) with neurosarcoidosis than with multiple sclerosis [20].

In patients with leptomeningeal involvement, the CSF findings are as follows: 40-70% exhibit pleocytosis; 40-73% has an elevated protein; and 10-20% has a low glucose [4, 5, 20, 44]. The mean number of CSF lymphocytes reported in the patients with pleocytosis in one series was 78, with a range from 8-300 per mm^3^ [5]. Oligoclonal bands and an elevated IgG index are encountered in up to 53% [20, 45]. The oligoclonal bands are usually accompanied by an elevation in protein [9]. Cultures for bacteria, fungi, and mycobacteria must be sterile, and cytology should be negative for neoplasm. CSF is often normal with isolated facial palsies [8]. From subsets of retrospectively studied patients, it has been found that CSF ACE levels are elevated in 24-55%, and thus insensitive, but they may be highly specific (94-95%) [46].

Electroencephalography may reveal focal or generalized slowing, but it is usually normal unless patients are having seizures [4]. Electrodiagnostic testing may be useful in patients with suspected neuropathy or myopathy.

## PATHOGENESIS

The cause remains uncertain. Observations of outbreaks and clustering of disease suggests a common environmental exposure, infectious agent, or genetic predisposition in some [2, 47, 48]. A single gene has not been identified as being causative. However, familial clustering in 19% of affected African-American families and in 5% of Caucasian families as well as associations with class I HLA-A1 and -B8 and class II HLA-DR 3 in Caucasians [49, 50] and HLA-DRB1 and DQB1 [2] suggest a genetic predisposition. There may also be a role for vitamin D deficiency, which is more prevalent in African-Americans [51].

As mentioned, the histopathologic lesion is the non-caseating granuloma. It is thought that granuloma formation begins with exposure to an antigen and is followed by T-cell and macrophage activation *via* a classic major histocompatibility complex (MHC) II-mediated pathway. It is further mediated by T-helper cells and activated T cells. Macrophages and dendritic cells release cytokines and chemokines, including interferon γ, tumor necrosis factor ∝, and interleukin (IL) including IL-2, IL-6, IL-12, IL-15, IL-16, and IL-18. Other cells are then recruited to the site of granuloma formation and become activated [51].

Also in favor of a driving T-helper response, there is evidence of decreased expression of natural killer cell inhibitory receptors on CD8+ T cells. This scenario possibly causes impairment in controlling the cell-mediated response [51]. Following accumulation of mononuclear inflammatory cells in the affected tissues, macrophages tightly aggregate and differentiate into epithelioid histiocytes and multinucleated giant cells. CD-4 and CD-8+ lymphocytes and some B cells form a rim around the granuloma. Subsequently, the inflammatory nodule becomes encased in fibroblasts, mast cells, collagen fibers, and proteoglycans, forming a destructive region of fibrosis through an incompletely understood process.

## TREATMENT

### Corticosteroids

In all forms of neurosarcoidosis, corticosteroids are typically the first line of treatment. There are no clinical trials that dictate specific doses or dosing schedules. Typically oral prednisone is dosed at 40-80 mg per day. In more severe CNS disease, intravenous corticosteroids may be administered. Benefits vary from substantial improvement – about half – to no benefit [5], and some patients succumb despite treatment [10]. Results of corticosteroid therapy from large series are summarized below.

In the study by Lower, *et al.,* 61 patients with neurosarcoidosis received corticosteroids. Those with isolated facial nerve palsies had an excellent response. Forty-eight others were treated with corticosteroids alone. Four died, but one death was unrelated to neurosarcoidosis. Thirty went on to have subsequent therapies and fourteen (29%) were treated long-term with prednisone only [3]. Chapelon, *et al.,* treated 31 patients with corticosteroids [6]. Ten patients completely recovered. Three required subsequent therapies with chlorambucil, methotrexate, or cyclosporine, and recovered fully. Ten patients treated with corticosteroids alone improved but had persistent deficits.

Of the 28 patients with neurosarcoidosis reported by Wiederholt, *et al.,* corticosteroids were used in nine. One died; four improved or recovered; and four were unchanged. Of note, 14 patients were not treated with immunosuppressive agents, and two received only anticonvulsants. Of these, at least ten improved or recovered [7]. In the series of 33 patients reported by Stern, *et al.,* 25 were known to receive prednisone. Of these, there was improvement or resolution in at least 19. One died. The response was unknown in several, and there was no improvement in at least one other. Some of the patients who benefited also had persistent deficits [4].

Zajicek *et al.* reported 48 patients treated for at least 18 months. Thirty-four received oral corticosteroids with or without IV methylprednisolone boluses. Twenty-nine percent improved or stabilized, while 71% worsened and then received other immunotherapy or cranial irradiation [20]. Joseph *et al.* treated 30 patients in a similar fashion with similar outcomes [9].

Pawate *et al.* treated 38 patients with either oral or IV corticosteroids followed by maintenance oral steroids. Eleven received corticosteroids alone, and the rest were offered other immunotherapies. In general, patients with bilateral optic neuropathies and those with widespread parenchymal or meningeal disease did poorly [11]. Scott *et al.* treated 19 patients with corticosteroids alone (60-80 mg/day). Eight of 19 were successfully weaned off in 2-3 years. Overall, 35% improved; 55% stabilized; and 10% worsened. Patients with more severe CNS involvement were treated with combination therapies (discussed in next section) [52].

The precise mechanisms of action of corticosteroids in treating neurosarcoidosis are not known, but presumably benefit is due to the known anti-inflammatory and immuno-modulating effects. These include interference with the function of leukocytes and fibroblasts, inhibiting access of leukocytes to inflammatory sites, and suppressing the myriad of humoral factors such as cytokine release involved in granuloma formation [53]. In particular, glucocorticoids antagonize the differentiation of macrophages and inhibit their functions. They block the release of cytokines such as IL-1, IL-6, and tumor necrosis factor ∝ [53]. Corticosteroids also inhibit T cell activation by binding to T cell receptors. In contrast, the immunosuppressive effects of corticosteroids affect B cells less, though they do reduce serum immunoglobulin levels, at least transiently.

Patients who are treated with corticosteroids should receive appropriate prophylaxis for osteoporosis and be monitored for hyperglycemia, cataract formation, psychiatric and infectious complications. They should eventually be treated with the lowest dose required to maintain benefit. Morning dosing should be used. Patients also need to be aware of the risk of developing cosmetic changes. Adrenal insufficiency may occur, especially if the dose is rapidly tapered or abruptly discontinued. Patients may also be at increased risk for peptic ulcer diseases, especially if they receive concomitant non-steroidal anti-inflammatory drugs [53].

## SECOND LINE TREATMENT OPTIONS

### “Steroid-sparing” Immunosuppressive Drugs

Second-line treatments may include methotrexate, cyclosporine, azathioprine, cyclophosphamide, chlorambucil, chloroquines, and mycophenolate. Stern, *et al.,* treated six patients with cyclosporine in a 12-month open label trial. They were able to lower baseline corticosteroid doses by 30-58%. It appeared that cyclosporine was beneficial in some patients, but others worsened despite a combination of cyclosporine and corticosteroid therapy [54]. Cyclosporine inhibits the CD4 cell immune response and IL-2 release. It can be started at 4 mg/kg/day in divided doses and requires careful monitoring of trough levels and for adverse events including hypertension, renal failure, hypomagnesemia, and neurotoxicity.

In the series by Lower, *et al.,* methotrexate, a dihydrofolate reductase inhibiter, successfully treated 61% (17 of 28) at doses of 5-15 mg/week. Liver biopsies, performed in 13 patients, did not reveal hepatotoxic changes. One patient developed neutropenia, and one had refractory nausea. Patients treated with methotrexate also require monitoring for pulmonary and renal toxicity [3]. Administration of folinic acid may reduce toxicity. Intermittent IV cyclophosphamide (starting at 500-700 mg every two weeks) “controlled the disease” in 9 of 10 treated patients, and it was well-tolerated. Most were treated for six or more months and had been previously treated with prednisone and methotrexate. Some remained on low-dose prednisone [3]. Cyclophosphamide is a potent immunosuppressant alkylating agent that cross-links DNA and RNA inhibiting protein synthesis. It requires careful monitoring for infection, hemorrhagic cystitis, as well as bone marrow and other toxicities.

Agbogu, *et al.,* reported their retrospective experience with various medications for corticosteroid-refractory neurosarcoidosis. Treatments included azathioprine (50-200 mg/day in 12 patients), cyclosporine (50-980 mg/day in 15 patients), cyclophosphamide (200 mg/day in three patients), chlorambucil (8-16 mg/day in two patients), and methotrexate (10-20 mg/week in two patients). Of 26 patients, six (23%) had improvement while receiving alternative medications, and 35% were stabilized. Fifteen percent did not respond to alternative therapies. Some patients took different drugs alone or in combination. Specific treatment recommendations cannot be made based on this study, but the authors did conclude that the choice of alternative therapy should be determined “in part, by its potential adverse effects” [55]. In the study, two patients treated with azathioprine developed neutropenia and one developed abnormal liver function tests. None had pancreatitis. Allergic reactions, which occur in 10% of patients treated with azathioprine, were not reported. One developed neutropenia with cyclophosphamide. Two developed renal dysfunction on cyclosporine. The only patient treated with chlorambucil developed leukopenia.

Scott *et al.* treated 26 patients with perceived severe CNS involvement with corticosteroids and steroid-sparing agents including methotrexate (n=18, 7.5-15 mg weekly), azathioprine (n=9, 150-200 mg daily), or monthly cyclophosphamide (n=9, 600-800 mg/m^2^ for 3-6 months). Overall, 18 (69%) improved; 4 (15%) stabilized; and 4 (15%) worsened including two deaths in patients with chronic meningitis [52].

Sharma performed a retrospective study in 12 patients with neurosarcoidosis treated with chloroquine or hydroxychloroquine for 6 to 21 months. These antimalarial drugs are used for treatment of connective tissue diseases and have an uncertain mechanism of action in sarcoidosis. Hydroxychloroquine prevents insulin degradation in the liver and suppresses gluconeogenesis, increasing peripheral utilization of glucose; so, it is particularly attractive to use in patients also treated with corticosteroids. Sharma reported that 10 of 12 patients had either stabilization or improvement in neurologic symptoms. These patients had previously failed to respond to corticosteroids or developed corticosteroid side-effects [56]. Patients must be monitored for ocular toxicity, but such effects were not observed.

### Biological Agents

Based on the presence of increased cytokine activity in the inflammatory response, especially increased levels of tumor necrosis factor (TNF), treatment with TNF ∝ blockers has been utilized. Eighteen patients with ocular sarcoidosis were treated with either etanercept or placebo in a double-blind, randomized trial. No benefit was seen in this small study, and there were no severe adverse events [57].

In contrast, the results of an open label study using infliximab and mycophenolate mofetil were promising but not definitive. Patients with CNS sarcoidosis who had “failed” treatment with steroids were given infliximab, and six of seven were also treated with mycophenolate mofetil. There was no placebo group. All patients reported significant benefit, both symptomatically and with regard to reversal of neurological deficits or control of seizure activity. There was also universal benefit as detected by MRI lesion size or gadolinium enhancement. There were no serious adverse events. The TNF ∝ neutralizer was coupled with mycophenolate, based on standard practice which postulates that the combination of infliximab with an oral immunosuppressive agent prevents otherwise expected development of human anti-chimeric antibodies [58]. Hopefully, this treatment combination will be subjected to a controlled prospective study.

Infliximab treatment was also associated with improvement in small-fiber neuropathy with autonomic involvement in one patient [59]. Intravenous immunoglobulin was also thought to be beneficial in a single patient with sensorimotor axonal polyneuropathy from sarcoidosis [60].

### Cranial Irradiation

There have been a few anecdotal reports of benefit from cranial irradiation [40]. Of the two patients reported by Chapelon, *et al.*, one had chronic meningitis, psychiatric disturbances and seizures [6]. He was unresponsive to corticosteroids and methotrexate, but recovered after 200 rads. The other had hemiparesis, an extrapyramidal syndrome, and severe psychiatric features unresponsive to steroids, but improved dramatically after 6000 rads. There was also dramatic improvement in CT scan findings [6]. Radiotherapy (1.3-3.6 Gy/day for 3-24 weeks) was also said to be beneficial in one of three patients treated by Agbogu, *et al.* [55].

## Figures and Tables

**Table 1:** Neurologic Manifestations of Sarcoidosis

	Zajicek *et al. *[20] N=68	Joseph and Scolding [9] N=30	Pawate *et al.* [11] N=54	Delaney, *et al. *[5] N = 23	Stern, *et al.* [4] N = 33	Chapelon, *et al. *[6] N = 35	Oksanen [10] N = 50	Lower, *et al.* [3] N = 71	Wiederholt, Siekert [7] N = 28
Cranial neuropathy % (N)	34 (23)	28 (8)	23 (12)	48 (11)	73 (24)	34 (12)	42 (21)	70 (50)	64 (18)
Optic neuropathy	38 (26)	37 (11)	35 (19)	30 (7)	12 (4)	3 (1)	10 (5)	10 (7)	21 (6)
Endocrine/hypothalamic dysfunction	3 (2)	17 (5)	2 (1)	26 (6)	15 (5)	11 (4)	10 (5)	8 (6)	25 (7)
Other intracranial mass	--	--	--	35 (8)	0 (0)	--			
Seizures	--	10 (3)	17 (9)	22 (5)	0 (0)	14 (5)	18 (9)	7 (5)	18 (5)
Meningitis	12 (8)	22 (7)	--	26 (6)	18 (6)	40 (14)	8 (4)	40 (28)	--
Myelopathy	28 (19)	15 (5)	19 (10)	9 (2)	6 (2)	0 (0)	10 (5)	--	4 (1)
Peripheral Neuropathy	--	--	2 (1)	4 (1)	6 (2)	40 (14)	18 (9)	4 (3)	14 (4)
Myopathy	--	--	--	9 (2)	12 (4)	26 (6)	10 (5)	--	7 (2)
